# Factor structure and measurement invariance of the Patient Health Questionnaire-4 among the Chilean population

**DOI:** 10.1371/journal.pone.0304623

**Published:** 2024-05-31

**Authors:** Ximena Moreno, Francisco Moreno

**Affiliations:** 1 Facultad de Psicología y Humanidades, Universidad San Sebastián, Santiago, Chile; 2 Department of Mathematics and Computer Science, University of Santiago, Santiago, Chile; University of South Asia, BANGLADESH

## Abstract

**Background:**

The PHQ-4 is an ultrabrief screening test for depression and anxiety symptoms. The psychometric properties of this test among the population in Chile are unknown. This study was aimed to determine the factor structure of the PHQ-4 in the adult population in Chile, and to assess its measurement invariance across different groups.

**Methods:**

The study considered a nationally representative sample of 10921 people aged 18 and above, in Chile in 2021. Exploratory and confirmatory factor analysis were conducted, and configural, metric, scalar, and strict measurement invariance were assessed.

**Results:**

The two-factor structure of the PHQ-4 supported the two underlying constructs of depression and anxiety. This model explained 67% of the variance and had excellent fit (CFI: 0.9999; TLI: 0.9994; RMSEA: 0.0107; SRMR: 0.0022). Strict measurement invariance held across gender, age, area of residence, household income quintile, immigrant or host population, and indigenous or non-indigenous population (ΔCFI<0.01).

**Conclusion:**

The PHQ-4 can be used to assess depressive and anxiety symptoms in population studies, and as a screening test for depression and anxiety in public mental health programs in Chile.

## Introduction

Mental health conditions are among the leading causes of disability-adjusted life years (DALYs), corresponding to the sum of years lost due to premature mortality and disability from a specific cause of less than good health. The most recent Global Burden of Disease (GBD) study estimated that 4.9% of global DALYs, and 14.6% of global years lost to disability (YLDs), which are years spent in less than good health, can be attributed to mental disorders [1]. According to the GBD study, the contribution of mental health conditions to global DALYs has increased 1.8 percent points, between 1990 and 2019. It has been previously highlighted that the estimations of the GBD could underestimate the contribution of mental health conditions to DALYs [2, 3], because self-harm and suicide are classified as injuries, and premature mortality due to mental health diseases is excluded. A recent estimation that considered these causes, concluded that 16% of the global DALYs are due to mental disorders [3]. Depression and anxiety are the two most common mental disorders [1], and according to a systematic review, the prevalence of depression was 0.68 percent points higher (27.6% increase), and the prevalence of anxiety was 0.95 percent points higher (25.6% increase), during the first year of the COVID-19 pandemic [4].

Public health is aimed at improving and promoting the health status of individuals and groups of the population [5]. Measurements of health status are used to describe, analyze, design, and evaluate interventions [5]. Therefore, public health must assess the mental health status of the population, to determine needs, identify inequalities and gaps, evaluate policies, anticipate the demand for mental health services, and monitor changes across time. An important tool to measure population mental health are screening instruments, which should be brief and easy to apply, but also valid.

The Patient Health Questionnaire-4 (PHQ-4) is an ultrabrief scale, aimed at detecting anxiety and depression symptoms [6]. It includes the two questions of the Generalized Anxiety Disorder-2 [7], from the Generalized Anxiety Disorder-7 [8], and the two questions of the Patient Health Questionnaire-2 [9], from the Patient Health Questionnaire-9 [10], to assess major depressive episode [6]. It is widely used in public health research [11]. The psychometric properties of the PHQ-4 had been studied previously, mainly in Europe [12–16] and the United States [6, 17–21] but also in Asia [22, 23], Africa [24], and South America [25, 26]. Most of these studies have focused on specific groups of the population, such as university students or adolescents and young adults [2, 18, 23, 26], health service users [6, 13, 14, 27] or people with health conditions [15], but a few have considered nationally representative samples [12, 16, 25], one of them including migrants and refugees [19]. A two-factor structure has been found more frequently [6, 12, 13–18, 20, 22, 23, 25, 26], in accordance with the two constructs that the scale is aimed to measure. Nevertheless, the findings of two studies support a one factor structure [21, 24], which could reflect the ability to measure a more general construct of psychological distress and potential case of mental disorder [6]. Measurement invariance across gender and age groups has been reported by several studies [12, 13, 22, 25, 26]. This has also been observed across language groups among Hispanics in the United States [17, 21], and among host population, migrants, and refugees in Germany [19]. However, several of the previous studies considered particular groups of the population, in terms of age [23, 26], or cultural background [17, 21]. A study that compared population from different countries reported partial scalar measurement invariance by age, gender, and country [16].

The psychometric properties of the PHQ-4 have been scarcely studied in South America [25, 26]. This instrument has been used in population studies in Chile, particularly during the COVID-19 pandemic [27, 28]. However, its factor structure and measurement invariance are unknown in the Chilean context. It is important to find out if the results of this scale can be interpreted as an indicator of psychological distress, or if it can be assumed that both depression and anxiety symptoms can be analyzed separately. Also, it is necessary to determine if the results of this instrument can be compared across different groups of the population in Chile. The aims of this study are a) to determine the factor structure of the PHQ-4 in the adult population in Chile, and b) to assess its measurement invariance across gender, age, area of residence, socioeconomic indicators, and cultural background.

## Methods

This study is a secondary analysis of data from the Social Wellbeing Survey (SWS), carried out in 2021 by the Ministry of Social Development in Chile to measure the level of wellbeing of the population, as an input to evaluate and design social public policies [29].

### Participants

The SWS recruited a sample of 10921 people (42.2% men; 67.8% women), representative of the national, urban and rural, and regional population aged 18 or more years in Chile [29]. The mean age of the sample was 46.4 years (SD 17.7), with a range of 72 years. As observed in Table 1, most of the sample lived in urban areas (85.2%). With respect to level of education, 34.4% of participants had incomplete secondary education, 43.4% had complete secondary education, and 21.7% had an undergraduate degree or more. The distribution of household income quintile was 20.7% in the lowest income quintile (I), 23.4% in quintile II, 21.2% in quintile III, 19.7% in quintile IV, and a lower proportion of people (15.1%) in the highest income quintile (V). A total of 541 participants (5.0%) were immigrants, defined as foreign born. A total of 1561 (14.3%) people declared they belonged to an indigenous nation or group.

**Table 1:** Sociodemographic characteristics of the sample.

	n	%
Gender		
Men	4613	42.2
Women	6308	67.8
Age		
18–29	2488	22.8
30–59	5553	50.8
60 or more	2880	26.4
Area of residence		
Urban	9307	85.2
Rural	1614	14.8
Level of education*		
Incomplete secondary education	3752	34.6
Complete secondary education	4736	43.6
Undergraduate degree or more	2366	21.8
Household income quintile		
I	2256	20.7
II	2557	23.4
III	2310	21.2
IV	2148	19.7
V	1650	15.1
Immigrant**		
Yes	541	5.0
No	10229	95.0
Indigenous nation or group		
Yes	1561	14.3
No	9360	85.7

*67 participants that answered “do not know” are not included.

**151 participants that answered “do not know” are not included.

### Procedures

The SWS employed telephone interviews to collect the data, between April and May 2021, carried out by trained interviewers [30]. The modules of the questionnaire included questions about sociodemographic characteristics, health status, social relationships, and income, among others. The answers were registered in a survey software, both in tablets and personal computers. The database was validated by the Microdata Center of Universidad de Chile, and the Ministry of Social Development [30].

### Measurements and variables

The PHQ-4 is a four-item questionnaire to assess depression and anxiety symptoms [6]. The question employed was: “Over the last 2 weeks, how often have you been bothered by the following problems?”

The first two items from the PHQ-9 [7], referred to depressive symptoms:

(1) “Little interest or pleasure in doing things” (PHQ1), and
(2) “Feeling down, depressed, or hopeless” (PHQ2).

The other items from the GAD-7 [9], assessing anxiety symptoms, were:

(3) “Feeling nervous, anxious or on edge” (GAD1), and
(4) “Not being able to stop or control worrying” (GAD2).

Participants were asked to respond, considering a 4-point Likert scale with the alternatives “never”, “several days”, “more than half the days”, and “nearly every day”, scored from 0 to 3 points. Hence, the measurement scale for these items is ordinal.

Age was categorized into three groups: 18–29, 30–59, and 60 or more years. Area of residence was a dichotomic variable with the categories “urban” and “rural”. Two variables were used as indicators of socioeconomic position. The first one, level of education, considered the categories “incomplete secondary education”, “complete secondary education”, and “undergraduate degree or more”. The other was household income quintile, where the first quintile corresponds to the lowest income quintile, and the last one is the highest income quintile. The indicators of cultural background were two dichotomic variables: immigrant status, defined as foreign born (yes or no), and member of an indigenous nation or group (yes or no).

### Statistical analysis

We carried out descriptive analyses of the PHQ-4 items and scale, including mean scores and standard deviation for each item. We assessed the internal consistency with inter-item correlation, corrected item-total correlation and Cronbach’s alpha. We also calculated McDonald’s omega.

We conducted exploratory factor analysis (EFA) and confirmatory factor analysis (CFA) to determine the factor structure of the PHQ-4. We performed Bartlett’s test of sphericity to verify the adequacy of the data for these analyses. The result (*p*<0.001) confirmed that the items of the PHQ-4 were correlated. As suggested for large samples [31], we complemented the result of this test with the Kaiser-Meyer-Olkin (KMO) test. According to the result of the KMO (0.76), the data was adequate to perform EFA. Polychoric correlations were calculated, which is recommended for EFA with ordinal data [32, 33]. The factor estimation method was minimum residual (MINRES), which has no distributional assumptions [31], and is recommended for large samples, when the items have few ordered response categories, and the analysis is based on a polychoric matrix [34]. Previous studies support the use of oblique rotation when the latent factors are assumed to be correlated, which is the case in psychology and mental health scales [31, 35]. Considering that, we employed oblimin rotation. Although most studies have found a two-factor structure of the PHQ-4, a few studies have reported a one-factor model had a better fit [21, 24]. Therefore, we performed CFA to compare a one-factor model and a two-factor model. In this case, we used the maximum likelihood method. With respect to the fit of the models, it is difficult to obtain non-significant results from large samples with the chi-square (χ2) test [25]. Hence, the comparison of models was based on the Comparative Fit Index (CFI), Tucker-Lewis Index (TLI), Root Mean Square Residual (SRMR), and Root Mean Square Residua (RMSEA), considering the following fit criteria: CFI≥ 0.95, TLI≥ 0.95, SRMR≤ 0.06, and RMSEA ≤ 0.06 [36].

We performed multi-group CFA to assess measurement invariance of the PHQ-4 across gender, age, area of residence, educational level, household income quintile, immigrant status, and indigenous group. We considered each variable with four models fitted to the data with increasing constraints [37]:

- Configural invariance: Models were estimated using the same baseline model method, based on two factors.
- Metric invariance (also named weak): Constraining the factor loadings to be equal.
- Scalar invariance (strong): Constraining the factor loadings and the item intercepts to be equal.
- Strict invariance: Constraining the factor loadings, the item intercepts, and the residual variances to be equal.

We examined differences (Δ) in successive model fit measures to compare the models, considering ΔCFI ≤ 0.010 as the cutoff value [38]. We additionally considered ΔRMSEA ≤ 0.015, and ΔSRMR ≤ 0.03 as indicators of measurement invariance, for comparative purposes with previous studies. The analyses were performed with psych [39], lavaan [40], and semTools [41] packages in R [42].

### Ethical considerations

The authors of this study analyzed the public database of the SWS, which is anonymized and available from the website of the Ministry of Social Development in Chile. Hence, no ethical approval was required.

## Results

The mean score of the PHQ-4 was 3.2, with a standard deviation of 2.8. As observed in Table 2, the mean score of the items ranged from 0.61 to 0.93, with considerable dispersion. The highest correlations were observed between PHQ1 and PHQ2 (0.69), between PHQ2 and GAD1 (0.64), and between GAD1 and GAD2 (0.63). Corrected item-total correlations ranged between 0.54 and 0.67. The internal consistency of the scale, according to Cronbach’s alpha (0.79) was acceptable, and it did not improve if any of the items was deleted. The results of McDonald’s omega were consistent (0.72).

**Table 2:** Item description and internal consistency of the PHQ-4.

Item	Mean (SD)	PHQ2 correlation	GAD1 correlation	GAD2 correlation	Corrected item-total correlation	α if the item is deleted
PHQ1 (Little interest or pleasure in doing things)	0.90 (0.92)	0.69	0.52	0.48	0.57	0.75
PHQ2 (Feeling down, depressed, or hopeless)	0.80 (0.86)		0.64	0.57	0.67	0.70
GAD1 (Feeling nervous, anxious, or on edge)	0.93 (0.92)			0.63	0.62	0.73
GAD2 (Not being able to stop or control worrying)	0.61 (0.90)				0.54	0.77

**Fig 1:**
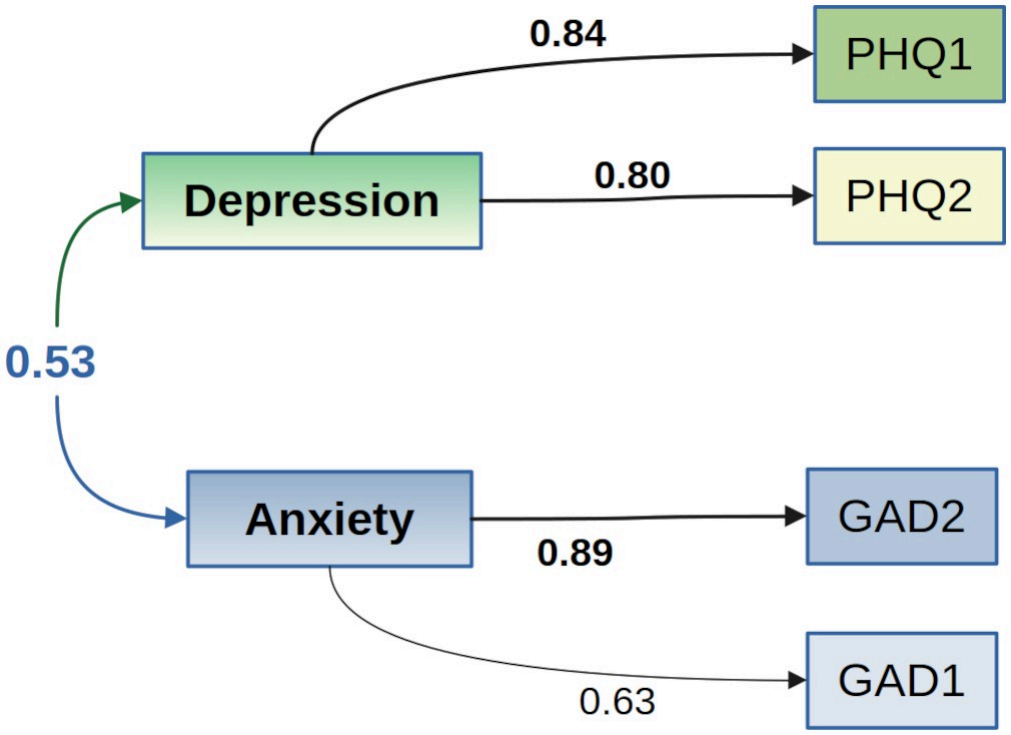
Two-factor model of the PHQ-4 (*n* = 10921).

The EFA showed one factor (depression, 36% of total variance) with high loadings on PHQ1 (0.84) and PHQ2 (0.80) and another factor (anxiety, 32% of total variance), with high loadings on GAD1 (0.89) and GAD2 (0.63). Both factors explained 67% of the variance. The correlation between both factors was 0.53 (Fig 1). According to the CFA, the two-factor model, based on different latent variables for depression and anxiety, explained a higher proportion of variance (67%) than the one-factor model (59%) encompassing the four PHQ-4 questions in one latent variable. Also, the fit indices of the two-factor model (CFI: 0.9999; TLI: 0.9994; RMSEA: 0.0107; SRMR: 0.0022) confirmed that this model had a better fit than the one-factor model (CFI: 0.9575; TLI: 0.8725; RMSEA: 0.1589; SRMR: 0.0393).

Table 3 summarizes the main statistics associated with each model and their differences (Δ) with respect to the previous test, with one less constraint. ΔSRMR and ΔCFI consistently are below their respective cutoff values (ΔSRMR below 0.03 and ΔCFI below 0.01), which suggests, in general, that the assumption of invariance holds. Only ΔRMSEA exceeds the cutoff value of 0.0150, when comparing the scalar and metric models grouped by age (0.0275), gender (0.0236), and education level (0.0165) and the metric and configural models for immigrant status (0.0203).

**Table 3:** Measurement invariance of the PHQ-4.

	Model fit indices				Model fit indices comparison			
Variable	χ^2^ (df)	CFI	RMSEA	SRMR	Δχ^2^	ΔCFI	ΔRMSEA	ΔSRMR
Gender								
Configural	4.58 (2)	.9998	.0154	.0021				
Metric	5.36 (4)	.9999	.0079	.0032	0.78	.0001	-.0075	.0011
Scalar	38.6 (6)	.9974	.0315	.0118	33.20	-.0025	**.0236**	.0086
Strict	52.82 (10)	.9966	.0280	.0140	14.26	-.0008	-.0035	.0023
Age group								
Configural	7.77 (3)	.9996	.0299	.0028				
Metric	18.9 (7)	.9991	.0216	.0091	11.11	-.0006	.0007	.0062
Scalar	108 (11)	.9926	.0491	.0168	88.81	-.0065	**.0275**	.0077
Strict	128 (19)	.9916	.0398	.0175	20.72	-.0010	-.0094	.0008
Area of residence								
Configural	2.39 (2)	1	.0059	.0019				
Metric	6.98 (4)	.9998	.0117	.0048	4.59	-.0002	.0057	.0029
Scalar	11.9 (6)	.9995	.0134	.0057	4.92	-.0002	.0017	.0010
Strict	25.9 (10)	.9988	.0166	.0086	13.19	-.0007	.0032	.0028
Educational level								
Configural	10.2 (4)	.9995	.0238	.0033				
Metric	31.5 (10)	.9984	.0281	.0112	21.33	-.0012	.0043	.0079
Scalar	103(16)	.9933	.0445	.0179	71.18	-.0050	.**0165**	.0067
Strict	164 (28)	.9896	.0422	.0203	61.29	-.0038	-.0024	.0024
Household income quintile								
Configural	3.9 (5)	1	0	.0021				
Metric	16.6 (13)	.9997	.0113	.0089	12.75	-.0003	.0113	.0069
Scalar	49 (21)	.9978	.0247	.0121	32.40	-.0019	.0134	.0031
Strict	110 (37)	.9943	.0301	.0172	61.17	-.0035	.0054	.0051
Immigrant/host population								
Configural	3.05 (3)	1	.0021	.0021				
Metric	19.8 (7)	.9990	.0224	.0059	16.78	-.0010	**.0203**	.0038
Scalar	38.8 (11)	.9979	.0264	.0069	19.02	-.0012	.0039	.0011
Strict	46 (19)	.9979	.0198	.0067	7.34	.0001	-.0065	-.0002
Indigenous/non indigenous								
Configural	8.74 (2)	.9995	.0249	.0035				
Metric	9.71 (4)	.9996	.0162	.0046	.96	.0001	-.0087	.0011
Scalar	17.4 (6)	.9991	.0187	.0058	7.73	-.0004	.0025	.0012
Strict	19.5 (10)	.9993	.0132	.0058	2.04	.0002	-.0055	.0000

Values of difference above the cut-off are in boldface.

## Discussion

This study assessed the factor structure of the PHQ-4, and its measurement invariance across gender, age, area of residence, socioeconomic indicators, and cultural background, among the adult population in Chile. Our results supported a two-factor structure, with one factor encompassing depressive symptoms, and the other, anxiety symptoms. Also, we could determine a strict measurement invariance of the test across different groups of the population in Chile.

Our results are concordant with most previous research, carried out at different periods and regions, that suggested a two-factor structure of the PHQ-4 [6, 12–16, 17, 18, 20, 22, 23, 25, 26]. It can be assumed that the instrument measures two latent variables, corresponding to depression and anxiety symptoms. On the other hand, as previously reported, we observed a high correlation between items of the different subscales (PHQ-2 and GAD-2). The co-occurrence of depression and anxiety is considerable, and it is associated with higher severity and less favorable clinical course [6, 43]. This stresses the importance of the PHQ-4 as a screener for depression and anxiety in population studies and in clinical practice [6]. In Chile, depressive disorders have received more attention from mental health research [44–47] and public policies [48]. Public programs of mental health include screenings for depressive symptoms, but anxiety symptoms are not routinely assessed. Nevertheless, although the diagnostic accuracy of the PHQ-2 to detect possible cases of depression has been studied in Chile [49–51], the sensitivity and specificity of the GAD-2 to screen for anxiety disorders are not known. International evidence suggests an acceptable diagnostic accuracy of the GAD-7 [6, 52], but this should be addressed by future research in Chile. If the evidence supports the use of the GAD-2 in clinical practice, the inclusion of the PHQ-4 as a screening tool in primary care could improve the detection of anxiety disorders and of comorbidity of depression and anxiety among the population in Chile.

According to the difference in CFI, which is the most widely used and empirically best supported fit index for the estimation method used [38], our findings support the measurement invariance of the PHQ-4 across different groups of the population in Chile. Other studies have considered the difference in SRMR and RMSEA. In our study, the differences in SRMR were at least ten times below the respective cutoff point, and most of the differences in RMSEA were below the recommended cutoff for metric measurement invariance [38, 53]. In the cases of differences in RMSEA above the cutoff point, we examined the specific models to determine the categories that explained these values [54]. In the case of gender, educational level and immigrant status, men, people with secondary education, and immigrant and host population, showed differences in both items about depressive symptoms. There were also differences in the measurements of anxiety symptoms (GAD2) among people with secondary education and older adults. Relaxing the respective constraints, the models reached better fits. A previous study reported partial scalar measurement invariance across gender, age, and country of residence in Europe [16]. However, most studies that have reported measurement invariance of the PHQ-4 do not consider the difference in RMSEA [12, 17, 21, 23, 25]. Another study used higher cutoffs for the difference in RMSEA [22], in which case the difference in RMSEA that we found would indicate measurement invariance in every case.

Although we have found measurement invariance of the PHQ-4 across different groups of the population in Chile, future research should address additional questions raised by our findings. First, it is necessary to assess the performance of this tool in clinical settings, to determine its diagnostic accuracy and its utility as a screening test for depression and anxiety in health care programs. Particularly, as discussed above, the sensitivity and specificity of the GAD-2 should be determined. Second, our results were obtained in the context of the COVID-19 pandemic. We observed measurement invariance across multiple groups, but we can only hypothesize that these results will be consistent with measurements obtained in a non-pandemic period. Therefore, it is important to conduct further analyses with data collected in subsequent studies, to verify this hypothesis. Finally, it would be valuable to conduct international comparisons, both across countries of Latin America that share linguistic and sociocultural background, and between Chile and countries of other regions of the world. The findings of a study that compared the factor structure of the PHQ-4 across European countries suggest that the measurement invariance is only partial across countries [16], but this should be determined by future studies, if comparable data is available.

This study has some limitations. The data collection was carried out via telephone interviews. It is unclear if the results obtained through this kind of interview are comparable to those obtained in a face-to-face assessment. Remote interviews are becoming more frequent in health research [55], but mental health screenings in public health programs in Chile are conducted in person. Response rate could differ [56], and certain groups could be more likely or not to report mental health symptoms to unknown interviewers [57]. Hence, our findings should be interpreted taking this into account. Also, the data analyzed in our study was collected during the COVID-19 pandemic. Studies carried out in this period in other countries showed similar results [22, 23], but there is no previous study about the psychometric properties of the PHQ-4 in Chile before this period. Therefore, as previously mentioned, future research should determine if the results obtained in this study hold in a non-pandemic context in Chile. Additionally, the number of items in the PHQ-4 is limited, which resulted in a reduced number of items per factor. It is usually recommended to hold at least three items per factor to obtain stable and robust estimates [34, 58]. Also, the ratio of the number of items to the number of factors affects overdetermination [59]. Other factors that interact with the number of items per factor are sample size and communalities [34], which suggests that a limited number of factors could be compensated by a large sample size and moderately high communalities. In our study, we analyzed data from a large sample, and the communalities were high (between 0.66–0.79) for three variables, and moderate for another one (0.41). Among the strengths of this study, we analyzed a sample representative of the population in Chile, which included diverse groups. We were able to compare the factor structure of the PHQ-4 across different groups of the population, including immigrants and indigenous groups, which correspond to 7.5% and 12.8% of the population, respectively [60, 61]. We also analyzed and reported strict measurement invariance, which is not usual in studies about the psychometric properties of the PHQ-4.

## Conclusions

We have analyzed the factor structure of the PHQ-4, and assessed its measurement invariance in a national survey representing the Chilean population in 2021, including 10921 observations. The underlying constructs, based on the factors of depression and anxiety, showed measurement invariance by gender, age, area of residence, education level, household income quintile, being immigrant or not, and being indigenous or not. This ultrabrief test can be used to assess depressive and anxiety symptoms in population studies, and as a screening test for depression and anxiety in public mental health programs in Chile.
