# Correction: Factor structure and measurement invariance of the Patient Health Questionnaire-4 among the Chilean population

**DOI:** 10.1371/journal.pone.0306287

**Published:** 2024-06-25

**Authors:** The PLOS ONE Staff

The funding statement for this paper is incorrect. The publisher apologizes for the error. The correct funding statement is as follows: This work was supported by the Vicerrectoría de Investigación y Doctorados de la Universidad San Sebastián - Fondo USS-FIN-24-APCS-10.
